# Special Issue: “Innate Immune Sensing of Viruses and Viral Evasion”

**DOI:** 10.3390/v13040567

**Published:** 2021-03-26

**Authors:** Renate König, Carsten Münk

**Affiliations:** 1 Host-Pathogen Interactions, Paul-Ehrlich-Institut, 63225 Langen, Germany; 2 Clinic for Gastroenterology, Hepatology, and Infectiology, Medical Faculty, Heinrich-Heine-University Düsseldorf, 40225 Düsseldorf, Germany

In this Special Issue, a wide variety of original and review articles provide a timely overview of how viruses are recognized by and evade from cellular innate immunity, which represents the first line of defense against viruses. The success of the immediate response relies on the recognition of invariant features encoded by viruses termed pathogen-associated molecular patterns (PAMPs) and by specialized sensors called pattern recognition receptors (PRRs). In the review by Singh et al., the reader is provided with a broad overview of the innate sensing of viruses by diverse PRRs. The authors discuss recent progress in the understanding of the consequences of innate sensing on the central nervous system (CNS), a tissue that can be severely damaged by infections of diverse viruses [1].

The consequence of this surveillance network and the downstream pathway activation is the secretion of cytokines and type I interferons (IFNs). Schwanke et al. provide an in-depth review on the master regulator of the type I interferon response, IRF3, and the IFNβ enhanceosome [2]. They present an extensive array of host or viral modulators of IRF3 activity, promoting or negatively regulating the activity upon viral stimulation. The type I IFN pathways result in the expression of interferon-stimulated genes (ISGs). A subset of ISGs possess a direct antiviral activity. These so-called restriction factors are in most cases also intrinsically expressed in some cell types without innate signaling. An example of the diversity of these factors attacking viral replication and counteraction response of the virus can be found in the review on human cytomegalovirus [3]. Large-scale screening approaches are useful tools to identify novel sensors or modulators of the innate pathways or novel antiviral ISGs expanding our knowledge on antiviral responses. Krey et al. present a detailed snapshot on such global approaches to dissect the antiviral innate immune landscape [4].

Based on the nature of their nucleic acid and entry pathways, viruses are, in general, either sensed by RNA or DNA sensors. Retroviruses, including the Human Immunodeficiency virus, are RNA viruses that reverse transcribe their RNA genome into DNA. Different aspects of RNA or DNA sensing in retroviral infections are highlighted in one review [5] and one original article [6] and a review on the distantly related endogenous retrotransposons [7]. The recognition of DNA viruses was enigmatic until the recent discovery of several DNA sensors. Poxviruses are an example of DNA viruses that express a plethora of viral antagonists that block sensing by DNA sensors, such as cGAS or DNA-PK and, interestingly, also by RNA sensors [8]. In contrast, Hepatitis B virus may evade recognition by shielding the DNA within the viral capsid, although naked HBV DNA elicits a strong immune response in primary myeloid cells mediated by cGAS/STING [9]. Hepatitis D Virus (HDV), causing the most severe form of viral hepatitis, however, activates strong IFNβ/λ responses in hepatocytes. Still, counteraction strategies, e.g., hiding its viral RNAs, are discussed [10]. These publications exemplify the fact that viruses have evolved multiple ways to dampen the host IFN response by interfering, disrupting, or evading specific host regulators, both up- and downstream of IFN induction. The diverse viral escape pathways are described in the following reviews [7,8,10,11,12,13] and one original article on herpes-viruses [14]. A recent discovery stems from the observation that DNA sensing by the cGAS/STING pathway can be triggered by certain RNA viruses. Zhu et al. describe an unexpected strategy of flaviviruses to antagonize this DNA sensing pathway [12]. Im-portant players in recognizing and sensing RNA viruses are described for HDV [10] and Influenza virus [15]. Additionally, different strategies for RNA viruses to coun-teract innate responses are presented in original or review articles for coronaviruses [16], flaviviruses [17], orthomyxoviruses [11] and filoviruses [13]. Furthermore, unexpected pathways seem to play important roles in detecting and responding to viral infections. Eiermann et al. highlight the growing body of evidence concerning the role of stress granules in regulating antiviral innate responses and defense. They detail the crossroads of viral sensing and the stress response pathway [18]. Collectively, these reports exemplify the importance of the interplay between viruses and innate immune pathways and provide novel avenues for stimulating virus research and a perspective on possible novel targets for immune-mediated antivirals.
